# *MetaMed*: Linking Microbiota Functions with Medicine Therapeutics

**DOI:** 10.1128/mSystems.00413-19

**Published:** 2019-10-08

**Authors:** Han Zhao, Shaliu Fu, Yifei Yu, Zhanbing Zhang, Ping Li, Qin Ma, Wei Jia, Kang Ning, Shen Qu, Qi Liu

**Affiliations:** a Department of Endocrinology and Metabolism, Shanghai Tenth People’s Hospital, and Bioinformatics Department, School of Life Sciences and Technology, Tongji University, Shanghai, China; b Department of Ophthalmology, Ninghai First Hospital, Ninghai, Zhejiang, China; c School of Life Science and Technology, Huazhong University of Science and Technology, Wuhan, China; d R&D Information, Innovation Center China, AstraZeneca, Shanghai, China; e Department of Mathematics and Statistics, South Dakota State University, Brookings, South Dakota, USA; Pacific Northwest National Laboratory

**Keywords:** microbiota, metabolism, biosynthetic gene clusters, medicine therapeutics

## Abstract

Understanding how the human microbiome affects human health has consequences for treating disease and minimizing unwanted side effects in clinical research. Here, we present *MetaMed* (http://metamed.rwebox.com/index), a novel and integrative system-wide correlation mapping system to link bacterial functions and medicine therapeutics, providing novel hypotheses for deep investigation of microbe therapeutic effects on human health. Furthermore, comprehensive relationships between microbes living in the environment and drugs were discovered, providing a rich source for discovering microbiota metabolites with great potential for pharmaceutical applications.

## Abstract (precis)

Understanding how the human microbiome affects human health has consequences for treating disease and minimizing unwanted side effects in clinical research.

## OPINION/HYPOTHESIS

The importance of human microbiota in therapeutic outcomes is well recognized. Deciphering the effects of microbes on human health has become a challenging issue in clinical research (1). Microbes affect the human body through various pathways, such as by modulating host metabolism and immunity through the production of metabolites and the release of bioactive components, thereby influencing health status and disease occurrence and/or progression (1). Although the exact mechanism underlying such microbiota-host interactions is complex and remains largely underexplored (2), linking microbe metabolites to existing drugs or small molecules by taking advantage of the available annotation information on existing medicines will provide useful guidance and novel hypotheses to decipher the effect of microbes on human health (3).

To this end, we introduced *MetaMed* (*Metagenomics Medicine* mapping system), a novel and integrative system-wide correlation mapping system to link bacterial functions and medicine therapeutics. In this system, a well-defined similarity score between microbial metabolite entities and medicine entities is applied to link microbial functions and existing medicine therapeutics. We provide comprehensive and solid evidence that such a straightforward linking strategy can help to achieve accurate predictions of microbial effects on the human body. Furthermore, such predictions also help to derive hypotheses which will facilitate the discovery of microbiota metabolites with great potential for pharmaceutical applications.

## DEVELOPMENT OF *MetaMed*

If microbial metabolites and medicine molecules share similar structures and perturbation transcriptional profiles, they will likely have similar functions in the human body. In our study, we first collected 1,157 microbe biosynthetic gene clusters (BGCs) as well as their metabolites from MIBiG (4) and 8,226 drugs from DrugBank (5). It should be noted that MIBiG provides comprehensive annotations to indicate the laboratory conditions for metabolites produced by microbes. Other information related to the conditions of microbes producing certain metabolites in the human body is not available yet. Therefore, *MetaMed* is designed to present the potential ability for microbes to produce certain metabolites and encourage further investigations on them.

We then defined a similarity score by considering both the molecular structure (6) and perturbation transcriptional expression profiles (7) to connect microbe functions with available drug annotation information (see Text S1 in the supplemental material). We eventually obtained a total of 1,193,324 pairs for evaluating the potential connections between metabolites and drug entities, ranked by our well-defined similarity score. We leveraged KEGG pathway annotations to justify our proposed *MetaMed* score schema. We defined a metabolite and a drug sharing at least one disease-related KEGG pathway as holding a similar function, and such drug-metabolite pairs are taken as the positive samples in the benchmark, while the other metabolite-drug pairs which do not share any disease-related KEGG pathways are taken as the negative samples. We ranked all predicted drug-metabolite pairs by their *MetaMed* similarity scores and evaluated the prediction precision above a certain *MetaMed* score threshold. As a result, the precision is 100% when the *MetaMed* score is above 0.9 (32/32). The precision is 83% when the *MetaMed* score is over 0.8 (45/54) and 48% when it is over 0.7 (95/199). The precision drops to 34% when the *MetaMed* score is over 0.6 (41/735). These data indicate that a higher *MetaMed* score represents a higher possibility to achieve reliable metabolite-drug pair prediction results with similar drug-related functions as indicated by the KEGG pathway annotations (see Fig. 2a). Taken together, our *MetaMed* similarity score can effectively distinguish high-potential metabolite-drug linkages from negative ones, which can be applied to identify metabolites with potential therapeutic effects effectively.

10.1128/mSystems.00413-19.1 TEXT S1 Supplemental methods. Download Text S1, PDF file, 0.2 MB. Copyright © 2019 Zhao et al. 2019 Zhao et al. This content is distributed under the terms of the Creative Commons Attribution 4.0 International license.

**FIG 1:**
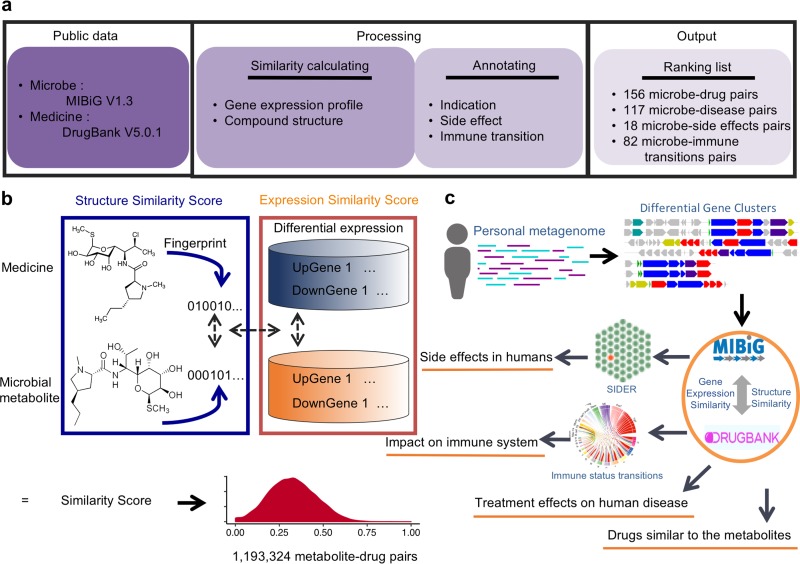
General pipeline for mapping microbe-medicine correlations. (a) Schematic overview of the data integration and processing steps. (b) Schematic depiction of the matching algorithm and score distribution. Through the similarity score calculated by the compound’s structure and gene expression profiles, a total of 1,193,324 microbe-drug pairs are presented in *MetaMed*. (c) A cartoon summary of the methods and integrated databases in *MetaMed*.

We further correlated the microbe functions with related drug effects by integrating various existing annotation information from MIBiG, LINCS, DrugBank, SIDER, etc. (8, 9) (Fig. 1; Text S1), resulting in a systematic mapping system, *MetaMed*, including the following links: microbe-drug, microbe-treatment indications, microbe-side effects, and microbe-immune status transition.

## NOVEL FINDINGS AND HYPOTHESES PROVIDED BY *MetaMed*

### *Actinobacteria* and *Ascomycota* are two phyla of microbes with metabolites enriched with potential therapeutic effect on human health.

**FIG 2:**
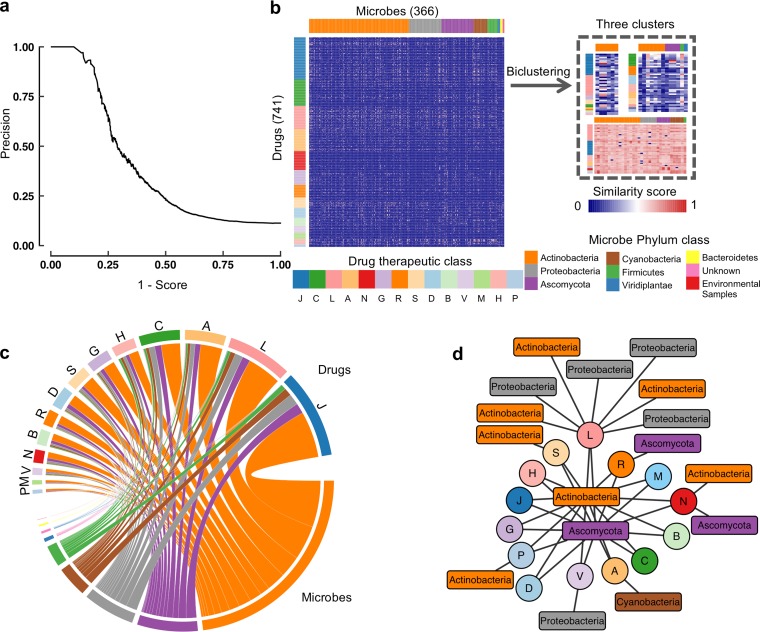
Global landscape of the entity relationships in *MetaMed*. (a) Precision-score curve for *MetaMed* predictions. The precision-score plot shows the precision above a certain *MetaMed* score justified by KEGG annotations. The *x* axis corresponds to 1 − *MetaMed* score. The *y* axis corresponds to the precision of metabolite-drug pair predictions above a certain score. (b) Biclustering results of the microbes and drugs predicted by the similarity score (cutoff = 0.6). Microbes are labeled by phylum, and single-letter codes for each drug follow the anatomic therapeutic classification system. Therapeutic classes include the following: H, systemic hormonal preparations, excluding sex hormones and insulins; V, various; B, blood and blood-forming organs; P, antiparasitic products; M, musculoskeletal system; L, antineoplastic and immunomodulating agents; G, genitourinary system and sex hormones; R, respiratory system; A, alimentary tract and metabolism; D, dermatologicals; J, anti-infectives for systemic use; S, sensory organs; N, nervous system; C, cardiovascular system. (c) Circular layout of the predicted connections between microbes and drugs (all connections with a similarity score of ≥0.6). Line widths correspond to the number of interactions. The diagram is organized by sorting the microbes clockwise (drugs counterclockwise) in order of decreasing number of connections. Single-letter codes for each drug follow the anatomic therapeutic classification system. (d) Subnetwork showing the microbe hubs and islands (rectangular nodes in the center and periphery, respectively) and their predicted interactions with drug subsets (circles). Each rectangular node represents a single microbe in the phylum (e.g., the upper center node labeled as *Actinobacteria* actually represents the microbe Streptomyces lusitanus, and the lower center node labeled as *Ascomycota* actually represents the microbe Aspergillus fumigatus).

To examine the global landscape of the entity relationships in *MetaMed*, we first applied the similarity score as a filter and organized the sets of drugs and microbial metabolites with a score cutoff of 0.6 by applying our developed biclustering algorithm (QUBIC for qualitative biclustering algorithm) (10) (Fig. 2b). QUBIC helps to clearly decipher the underlying pattern of microbe-drug relationships simultaneously from two perspectives. We found that microbes with similar phylum classes clustered together and correlated with certain drug categories with similar therapeutic effects. It is clear that the phylum *Actinobacteria* is enriched to be a microbe cluster with certain therapeutic indications (Fig. 2b). These clustered microbes with similar functional metabolites provide the most important resources of lead compound discovery. In addition, an overview of all the predicted connections above a similarity threshold (similarity score ≥0.6, Fig. 2c) is presented to cluster these drugs and microbes by therapeutic class and microbe phylum. In total, there were nine microbe categories at the phylum level (Fig. 2c). Our analysis revealed that microbes in *Actinobacteria* produce molecules with functions similar to anti-infective, antineoplastic, and immunomodulating drugs. This finding is consistent with previous studies (11) reporting that *Actinobacteria* are efficient producers of new secondary metabolites that exhibit a wide range of biologic activities, including antibacterial, antifungal, anticancer, antitumor, and anti-inflammatory activities. In addition, our analysis provided new clues for discovering potential bacterial lead producers with other therapeutic functions; e.g., microbes in *Ascomycota* and their enriched metabolites are potential sources with functions similar to those of cardiovascular system, anti-infective, antineoplastic, and immunomodulating drugs. We further defined the microbe hubs based on interactions with the greatest number of drug types, which were hypothesized to have the largest effects in humans. On the other hand, microbe islands were defined on the basis of interacting with the lowest number of drugs (Fig. 2d; Table S1).

10.1128/mSystems.00413-19.2 TABLE S1 List of microbe hubs and islands and their predicted interactions with drug subsets. The predicted connections above a similarity threshold (0.6) are presented to cluster the drugs and microbes by therapeutic class and microbe phylum. Microbe hubs are those microbes identified with the highest interaction number for drug types. Microbe islands are those microbes identified with the lowest interaction number for drug types. Download Table S1, XLSX file, 0.01 MB. Copyright © 2019 Zhao et al. 2019 Zhao et al. This content is distributed under the terms of the Creative Commons Attribution 4.0 International license.

### Certain microbes generate secondary metabolites as potential resources for new drug discovery.

**FIG 3:**
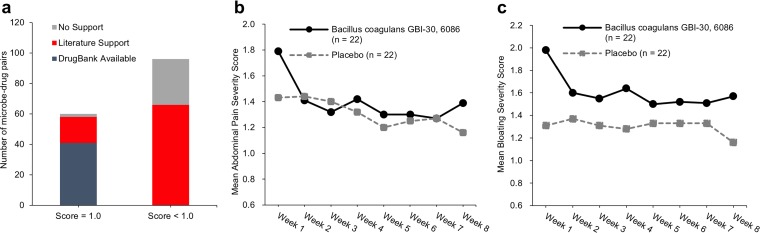
Validation of the *MetaMed* prediction results. (a) Most of the microbe-drug pairs can be validated by DrugBank or published literature. The first bar indicates that 60 pairs have a similarity of 1.0. The second bar indicates that 96 pairs have a similarity of <1.0. (b) Validation results of the effect of *B. coagulans* on IBS. The *x* axis corresponds to treatment weeks. The *y* axis corresponds to the mean abdominal pain scores and mean bloating scores. (c) Validation results of the effect of *B. coagulans* on IBS. The *x* axis corresponds to treatment weeks. The *y* axis corresponds to the mean bloating scores.

Based on the similarity cutoff of 0.9 for metabolite-drug pairs, *MetaMed* obtains 156 meaningful microbe-drug linkages (Table 1; Table S2). Among them, the similarity scores of 60 microbe-drug pairs were 1.0 (38.5%, 60/156), indicating that those drugs were originally isolated from these microbes or that these microbes can generate secondary metabolites that are exactly the same as the drugs. Of the 60 microbes, 41 (68.3%, 41/60) are already annotated to produce the corresponding drugs in DrugBank, while the remaining 17 (28.3%, 17/60) are novel identifications annotated to produce the corresponding drugs by *MetaMed*. For another 96 microbe-drug pairs, their similarity score was lower than 1.0 while still maintaining similarity over 0.9. Sixty-six of these microbes (68.8%, 66/96) are reported in the literature to have similar therapeutic indications as those of the corresponding drugs (Fig. 3a, Table 1, and Table S2). Our analysis reveals that these microbes can generate secondary metabolites that are similar to the corresponding drugs. We recommend that users focus on such pairs, since they are potential sources for new drug discovery.

**TABLE 1:** Summary of selected connections between microbes and drugs

*MetaMed* prediction	Score = 1.0		0.9 < score < 1.0		Total	
	Predicted	DrugBank validated	Predicted	Literature validated	Predicted	Validated
No. of microbe-drug linkages	60	41	96	66	156	107
No. of microbes with disease treatment effects	45	29	72	71	117	100

10.1128/mSystems.00413-19.3 TABLE S2 Predictions of microbe-drug linkages above the similarity score (0.9) and their validations by published literature. Novel identifications of microbes generating secondary metabolites similar to the corresponding drugs are marked gray. Download Table S2, XLSX file, 0.02 MB. Copyright © 2019 Zhao et al. 2019 Zhao et al. This content is distributed under the terms of the Creative Commons Attribution 4.0 International license.

### *MetaMed* identifies microbes with disease treatment effects.

Based on the 156 microbe-drug pairs identified, *MetaMed* directly predicts 117 microbes with disease treatment effects (Table 1; Table S3) by leveraging the drug annotation information (Fig. 1 and Text S1). Among them, 113 microbes exist in the environment, and they cannot survive in the gut. Their metabolites, however, can be ingested as compound treatments for the corresponding diseases. For certain microbial metabolites which are not available for ingestion, future studies are needed to determine their ingestion mechanisms. Among these 113 microbes, metabolites from 29 (25.7%, 29/113) are already annotated with their disease treatment effects in DrugBank as these metabolites are identical to the drugs. Another group of metabolites derived from 71 microbes (62.8%, 71/113) was also validated for disease treatment by literature-based evidence (Table 1; Table S3). In total, there is evidence to support that the secondary metabolites of 100 microbes (88.5%, 100/113) can be used to treat corresponding diseases. *MetaMed* predicts that another two endogenous microbes, i.e., Bacillus coagulans and Escherichia coli, which exist in the human gut, can treat irritable bowel syndrome (IBS) with a high score of 0.92 and 1.0, respectively. These predictions were validated by two clinical studies (12, 13). One study treated 44 IBS patients with either *B. coagulans* (*n *=* *22) or placebo (*n *=* *22) (12). Significant alleviation (*P < *0.01) in IBS symptoms, like abdominal pain and bloating, was achieved in IBS patients compared with control patients (Fig. 3b and c). Another clinical study revealed therapeutic effects of E. coli on IBS, especially in patients with altered enteric microflora (13).

10.1128/mSystems.00413-19.4 TABLE S3 Predictions of microbes with disease treatment effects above the score 0.9 and their validations by published literature. Novel identifications of microbes with similar therapeutic indications as those of the corresponding drugs are marked gray. Download Table S3, XLSX file, 0.03 MB. Copyright © 2019 Zhao et al. 2019 Zhao et al. This content is distributed under the terms of the Creative Commons Attribution 4.0 International license.

### *MetaMed* identifies microbes with side effects.

Based on these 156 identified microbe-drug pairs, *MetaMed* predicts 18 microbes with side effects (Fig. 1, Text S1, and Table S4). These microbes generally exist in the environment, and their secondary metabolites may have side effects in humans. Among these 18 microbes, metabolites from 5 microbes are already annotated with their side effects in SIDER (8) as these metabolites are identical to the drugs. Another group of metabolites derived from two microbes that are similar to drugs was also validated with potential side effects based on published evidence. *MetaMed* predicts that Streptomyces toxytricini produces the same side effects of oily stools, diarrhea, and dyspepsia. These predictions were successfully validated in a recent study (14) in which the clinical drug usage of lipstatin from *S. toxytricini* caused unpleasant side effects like oily stools, diarrhea, and dyspepsia. *MetaMed* also predicts that Streptomyces lividus has side effects on hearing, such as hearing impairment, high-frequency deafness, tinnitus, and total deafness. Interestingly, another study (15) also reported that lividomycin from *S. lividus* has side effects on the inner ear and that care must be taken to prevent ototoxicity. The potential mechanism of the side effects of these bacteria is that the secondary metabolites of these two microbes are similar to drugs with similar side effects.

10.1128/mSystems.00413-19.5 TABLE S4 Predictions of microbes with side effects above the score 0.9 and their validations by published literature. Download Table S4, XLSX file, 0.07 MB. Copyright © 2019 Zhao et al. 2019 Zhao et al. This content is distributed under the terms of the Creative Commons Attribution 4.0 International license.

### *MetaMed* predicts microbes with effects on immune transition.

*MetaMed* also predicts 82 microbes with effects on immune transition in human health (Table S5) leveraging recently published information on the drug-immune transition (9) (Fig. 1 and Text S1). Among them, 81 microbes exist in the environment, and they cannot survive in the human gut. The metabolites of these 81 microbes, however, can be taken as compounds with potential effects on the corresponding immune transition. Metabolites from 35 microbes (43.2%, 35/81) are already annotated with their impact on immune transitions (9) as these metabolites are identical to the drugs. For the only endogenous microbe identified by *MetaMed*, i.e., Bacillus coagulans, which exists in the human gut, *MetaMed* predicts that the microbe can increase CD4^+^ T cells in the spleen. Interestingly, one recent clinical study (16) demonstrated that administering *B. coagulans* significantly (*P* < 0.001) increases the population of CD4^+^ Foxp3^+^ T cells in the spleen.

10.1128/mSystems.00413-19.6 TABLE S5 Predictions of microbes with effects on immune transition based on the identified microbe-drug linkages above the similarity score 0.9 and their validations by published literature. Download Table S5, XLSX file, 0.06 MB. Copyright © 2019 Zhao et al. 2019 Zhao et al. This content is distributed under the terms of the Creative Commons Attribution 4.0 International license.

### Linking endogenous microbe alteration information with drug therapeutics serves as a potential strategy for drug combination prediction.

**FIG 4:**
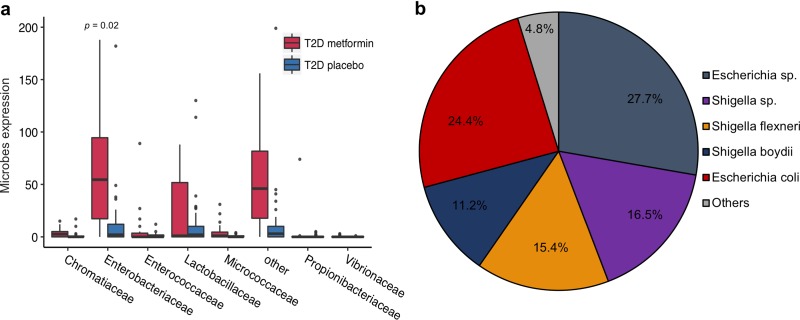
Validation of the *MetaMed* prediction of E. coli treating human T2D by MWAS. (a) E. coli at the genus level was significantly increased in the metformin group. The *x* axis corresponds to different microbes in the family, and “other” means that they cannot be identified at the family level. The *y* axis corresponds to the metagenomic read count. The *P* value of *Enterobacteriaceae* between read counts by taking the metformin group (red box plots) and read counts by taking the placebo group (blue box plots) is labeled at the top of the two box plots. (b) Most of the microbes in *Enterobacteriaceae* are E. coli. “Other” means the abundance is lower than 0.1%.

Alterations of endogenous microbes also affect disease treatment, and we further pointed out that linking endogenous microbe alteration information with drug therapeutics is a potential method for predicting the appropriate combination drugs for treating disease. We demonstrated this hypothesis by the following two cases. (i) Recently, two studies reported the involvement of gut microbes in regulating the efficacy of anti-CTLA-4 and anti-PDL1 cancer therapy (17, 18). The outgrowth of Bacteroides fragilis was associated with the efficacy of CTLA-4 blockade in the treatment of melanoma patients with ipilimumab (17). Although this is a correlation study, it is reasonable to speculate that the metabolites of B. fragilis help to improve the treatment efficacy of CTLA-4 blockade with ipilimumab. In our study, *MetaMed* found that glucosamine 1-phosphate (score = 0.73, ranking first among all B. fragilis metabolites) and sodium stibogluconate (score = 0.70, ranking second among all B. fragilis metabolites) have high similarity scores with B. fragilis metabolites. Interestingly, some studies report that sodium stibogluconate can be used to augment the blockade of CTLA-4 by ipilimumab (19). The potential immunotherapy effect of glucosamine 1-phosphate awaits further validation, although its potential immunosuppressive effects and anticancer activity were reported elsewhere (20, 21). (ii) We also analyzed the differentially expressed microbes in human disease from metagenome-wide association study (MWAS) data (22, 23). *Enterobacteriaceae* was the only bacterial family exhibiting highly increased expression in two similarly sized subgroups of women who had type 2 diabetes (T2D) with (*n* = 20) or without (*n* = 33) metformin treatment (Fig. 4a), and Escherichia coli was the most abundant genus in this case (Fig. 4b). In *MetaMed*, E. coli was identified to be correlated with T2D treatment effects, which is consistent with the MWAS result. *MetaMed* further identified that linaclotide is the drug most similar to E. coli metabolites (score = 1.00). We speculate that this drug could be a potential drug combination candidate for treating T2D, although no reports have been published to date. We investigated the next most similar drug, i.e., lixisenatide, which is similar to E. coli metabolites with a similarity score of 0.68. Surprisingly, this drug is reported to reduce blood glucose levels in patients with T2D (24) and is administered with metformin to improve treatment for T2D (25). In summary, this evidence, although limited, indicates that information linking clinical treatment-induced changes in endogenous microbes with drug annotation by *MetaMed* is a promising method for predicting combination drug effects.

## CONCLUSIONS

It should be noted that *MetaMed* utilized metabolites produced by microbial BGCs from MIBiG as the data source, which may lead to the exclusion of certain known drugs produced by microbes without BGC information and the experimental annotations of the corresponding microbe metabolites. For all 93 drugs reported to be produced by microbes in DrugBank, *MetaMed* identified 41 among them. Despite the limited annotations of MIBiG for BGCs, the MIBiG database provides comprehensive experimental annotations on how microbial BGCs produce certain metabolites under certain conditions, which is the primary concern of our study here. A microbe may have the genomic ability to produce a drug, but it happens only under certain treatment or laboratory conditions. Since the MIBiG database presents comprehensive annotations of the laboratory conditions for metabolites produced by certain microbes curated from published literature, it was selected as our data source. Other databases (26, 27) collect many more microbes however lack such annotations. Therefore, we believe that *MetaMed*, which is built based on the experimental validated annotations of microbe metabolites collected from the MIBiG database, can provide a valuable and reliable resource to the whole community.

To facilitate a comprehensive exploration of the systematic mapping of the microbiota functions with medicine annotations, we built the web system *MetaMed* V1.1 (http://metamed.rwebox.com/index) to allow users to browse various microbe functions and medicine annotation linkage information, as well as to calculate such linkages directly from personalized metagenomics sequencing data (Metapipe, https://github.com/adamtongji/metapipe). Novel findings and hypotheses can be obtained by analysis of such linkages, while future directions will be explored to investigate the causation between them by experimental study (28). In summary, *MetaMed* provides the first attempt to link microbiota functions with medicine therapeutics, providing novel perspectives and hypotheses for deep investigation of microbe therapeutic effects on human health. The aim of our study is to present a novel computational strategy to decipher microbe effects on human health, and we validated the identified relationships with only known literature evidences. We encourage to perform future experimental validation and investigations on these derived hypotheses or predicted results. Such knowledge will undoubtedly foster the successful application of nutritional microbiota-based therapeutics, enabling optimization of the efficacy of microbiota-interacting drugs in humans and facilitating the discovery of microbiota metabolites with great potential for pharmaceutical applications.

### Availability of data.

The data for correlation between microbes and drug, microbial information, and drug annotation are available at http://metamed.rwebox.com/index. The code for calculating such linkages between microbes and drug directly from personalized metagenomics sequencing data is available at https://github.com/adamtongji/metapipe.
