# Supplementary material for: MetaMed: Linking Microbiota Functions with Medicine Therapeutics
Source: mSystems. 2019 Oct 8;4(5):e00413-19. doi: 10.1128/mSystems.00413-19 (PMC6787565; doi:10.1128/mSystems.00413-19)
Supplement: TEXT S1 [file mSystems.00413-19-s0001.pdf]

MetaMed: Linking microbiota functions with medicine therapeutics

Supplementary Methods

1 BGC and drug annotation data collection

Host microbes information like biosynthetic gene clusters (BGCs) and their molecular products are downloaded from the *Minimum Information about a Biosynthetic Gene cluster* (*MIBiG*) specification (1) (version 1.3), which includes 827 microbes, 1,157 BGCs and 1,157 metabolites. We categorized these microbes into their phylum levels. The final data set included 1,157 BGCs and 1,157 metabolites for 827 microbes.

The drug data annotation information is obtained from *DrugBank* datasets (2) (version 5.0.1). We annotated 8,226 drugs from three aspects: (i) The indications of 8,226 drugs obtained from *DrugBank*, (ii) the common side effects of 332 drugs selected from *SIDER* (3) (version 4.1) and (iii) the immune status transitions of 1310 drug obtained from one recent study (4). We further labeled drugs with ATC classification system level 1 description. These therapeutic classes include: H, systemic hormonal preparations, excluding sex hormones and insulins; V, various; B, blood and blood-forming organs; P, antiparasitic products; M, musculoskeletal system; L, antineoplastic and immunomodulating agents; G, genitourinary system and sex hormones; R, respiratory system; A, alimentary tract and metabolism; D, dermatologicals; J, anti-infectives for systemic use; S, sensory organs; N, nervous system; and C, cardiovascular system. Finally, we curated the drug data set with 8,226 drugs, and annotated the drugs with indications, side effects, immune transitions and therapeutic classes.

2 Calculation of *MetaMed* entity relationships

We constructed a data set containing 1,157 microbe biosynthetic gene clusters (BGCs) as well as their metabolites from *MIBiG* (1) , and 8,226 drugs obtained from *DrugBank* (2). We defined a solid similarity score by considering both the molecular structure (5) and perturbation transcriptional expression profiles (6) to connect microbe functions with available drug annotation information.

To integrate the structural similarity and transcriptional expression similarity, we obtained the final similarity score of microbe-drug pairs by using the mean of structure similarity and transcriptional expression similarity, or using the structure similarity if the transcriptional expression is not available. 1,193,324 linkages were collected based on the structure similarity and finally 18,090 linkages were curated by considering both structure similarity and transcriptional perturbation profiles similarity.

Based on the microbe-drug pairs and drug annotations, the scores of microbes with disease treatment effects or side effects are defined directly as the similarity score of microbe-drug pairs. The score of microbes with the impacts on human immune transition is defined as:

Score(microbe-immune pair) = ***Score*** (microbe-drug pair) × ***Score***(drug-immune pair) [1]

2.1 Calculation of microbe-drug pairs structure similarity

To estimate the structural similarity between microbe metabolite and drug, we used the simplified molecular-input line-entry system (SMILES) (7) notation to describe these compounds, and calculated the similarity scores from SMILES notations. The SMILES notations of drugs and microbe metabolites are translated from their common name by *Chemspipy* (8). The structural similarity between two compounds is calculated as the

Tanimoto coefficient (9) of SMILES structural fingerprint by *Open Babel* (10). Given a compound A and a compound B, the Tanimoto coefficient for binary vectors is defined as:

$$\frac{N(A,B)}{N(A)+N(B)-N(A,B)} \quad [2]$$

where, N(A) and N(B) are the number of bits set on ('1' bits) in molecular fingerprints A and B respectively, and N(A,B) is the number of bits shared by A and B.

2. 2 Calculation of microbe-drug pairs transcriptional expression similarity

To predict transcriptional expression similarity of microbe-drug pairs, we obtained Drug-induced transcriptional profile changes determined from human cells lines from NIH *LINCS* program (6). We processed the whole data sets, which include more than 110,000 experiments tested over 8,000 compounds. We collected microbe transcriptional perturbation data by linking microbial metabolites to the *LINCS* compounds with the same compound names or same compound structures (SMILES notations). Then, we selected the up-regulated genes and down-regulated genes of the corresponding microbe metabolite from level 5 data of experiments in the same cell line at the same time point. We searched each metabolite for mimic drugs and their transcriptional expression similarity scores (cosine distance between metabolite and drug gene signatures) by *LINCS* API. Finally we filtered the mimic drugs by the same cell line and the same time point of input metabolite, and obtained the microbe-drug pairs and their similarity scores.

3 Global landscape of *MetaMed*

To examine the global landscape of the *MetaMed*, we first filtered the data by microbe-drug pairs similarity score. The low similarity score indicates a low correlation between drugs and microbes, and holds limit clues for identifying the real relationship between drugs and

microbes. We set the similarity cutoff of 0.6 for drug and microbe relationship for global analysis. Then we annotated the drug with ATC classification system level 1 description. We also categrized the microbe with phylum level. We obtained pairs from 366 microbes and 741 drugs in total, including 14 classes of drugs and 9 classes of microbes. By appliying our algorithm QUalitative BIClustering algorithm (11) using the *QUBIC R* packages (version 1.6.4 https://bioconductor.org/packages/release/bioc/html/QUBIC.html), we identified three potential biclusters, and they were visualized with heatmap.

4 Validation of the entity relationships in *MetaMed*

According to drug annotation information, we demonstrated the utility of the *MetaMed* from the following five aspects: meaningful microbe-drug linkings, microbes with disease treatment effects, microbes with side effects, microbes with the impacts on immune transition and finally identification of combination drug usage for disease treatment. We selected the pairs with similarity cutoff over 0.9 for validations.

We first validated the predicted metabolite-drug links. Two types of pairs are considered. If the similarity scores of microbe-drug pairs are 1.0, it indicates that these microbes can generate exactly the same drugs as the secondary metabolites. If the similarity score is lower than 1.0 while still maintains high similarity, it indicates that these microbes should have similar therapeutic indications as those of the corresponding drugs. For the first type, we found these microbes are already annotated to produce the corresponding drugs as indicated in *DrugBank*. For the second type of microbe-drug pairs, we validated them by published literature evidence.

For other identifications including microbes with disease treatment effects, microbes with side effects and microbes with the impacts on immune transition, we predicted the microbe impact on human directly. If some of the microbes exist in environment and they cannot survive in gut, we took their metabolites as the compound which may have impacts on human health. Then we validated them by availiable databases or published literature evidence.

To validate the combination drug usage, we validated the applications in disease treatment by data obtained from immune checkpoint therapy and Metagenome-wide association studies (MWAS) (12, 13). First, we combined *MetaMed* with two recent studies of the association of gut microbes in regulating the efficacy of anti-CTLA-4 and anti-PDL1 cancer therapy (14, 15). By investigation of the outgrowth microbes, we identified drugs with the similar functions as the secondary metabolites, and these drugs may be taken as the the potential drug combination for treatments. Next we combined *MetaMed* with MWAS and obtained raw sequence data (ERP002469) of healthy and T2D samples from MWAS. We processed the sequence data by *Metapipe* and obtained the abundance of OTUs. The differential OTU analysis was performed by *edgeR*(16). The outgrowth OTUs with *p-value* < 0.05 and logFC > 1.5 were selected as differential expressed OTUs. By such analysis, we predicted potential combination drug usage for T2D treatments.

5 Visualization of *MetaMed* entity relationships

Circos plots (17) are created using the *circlize R* package (version 0.0.7 https://github.com/jokergoo/circlize). Network diagrams were produced using *Cytoscape* (18).

All other plots are created using the *R* statistical package.

6 Building *MetaMed* platform

We built the web system *MetaMed* V1.1 (http://metamed.rwebox.com/index) for browsing various microbe functions and medicine annotations linkage information, as well as calculating such linkages directly from personalized metagenomics sequencing data (*Metapipe*, https://github.com/adamtongji/metapipe).

Metapipe can analyze two types of human sequence data, i.e., WGS data and 16S rRNA data. For the WGS data, we set the reference sequences from biosynthetic gene clusters (BGCs) sequences of microbes. The mapping step was performed by *Bowtie 2* (19) and *Samtools* (20). The output includes microbes and their expression estimated by read counts. Users can filter the microbe results from *Metapipe* by setting the cutoff of minimum mapped read counts. For the 16S rRNA data, *Metapipe* uses *QIIME* (21) to estimate the microbe abundance and obtains the result table including microbes and their corresponding estimated abundance information. Users can filter the microbe results from *Metapipe* by setting the abundance cutoff. The output of *Metapipe* can be directly used in the *MetaMed* online platform.

References

1. **Medema MH, Kottmann R, Yilmaz P, Cummings M, Biggins JB, Blin K, de Bruijn I, Chooi YH, Claesen J, Coates RC, Cruz-Morales P, Duddela S, Düsterhus S, Edwards DJ, Fewer DP, Garg N, Geiger C, Gomez-Escribano JP, Greule A, Hadjithomas M, Haines AS, Helfrich EJN, Hillwig ML, Ishida K, Jones AC, Jones CS, Jungmann K, Kegler C, Kim HU, Kötter P, Krug D, Masschelein J, Melnik AV, Mantovani SM, Monroe EA, Moore M, Moss N, Nützmann H-W, Pan G, Pati A, Petras D, Reen FJ, Rosconi F, Rui Z, Tian Z, Tobias NJ, Tsunematsu Y, Wiemann P, Wyckoff E, Yan X, Yim G, Yu F, Xie Y, Aigle B, Apel AK, Balibar**

CJ, **Balskus EP**, **Barona-Gómez F**, **Bechthold A**, **Bode HB**, **Borriss R**, **Brady SF**, **Brakhage AA**, **Caffrey P**, **Cheng Y-Q**, **Clardy J**, **Cox RJ**, **De Mot R**, **Donadio S**, **Donia MS**, **van der Donk WA**, **Dorrestein PC**, **Doyle S**, **Driessen AJM**, **Ehling-Schulz M**, **Entian K-D**, **Fischbach MA**, **Gerwick L**, **Gerwick WH**, **Gross H**, **Gust B**, **Hertweck C**, **Höfte M**, **Jensen SE**, **Ju J**, **Katz L**, **Kaysser L**, **Klassen JL**, **Keller NP**, **Kormanec J**, **Kuipers OP**, **Kuzuyama T**, **Kyrpides NC**, **Kwon H-J**, **Lautru S**, **Lavigne R**, **Lee CY**, **Linquan B**, **Liu X**, **Liu W**, **Luzhetskyy A**, **Mahmud T**, **Mast Y**, **Méndez C**, **Metsä-Ketelä M**, **Micklefield J**, **Mitchell DA**, **Moore BS**, **Moreira LM**, **Müller R**, **Neilan BA**, **Nett M**, **Nielsen J**, **O'Gara F**, **Oikawa H**, **Osbourn A**, **Osburne MS**, **Ostash B**, **Payne SM**, **Pernodet J-L**, **Petricek M**, **Piel J**, **Ploux O**, **Raaijmakers JM**, **Salas JA**, **Schmitt EK**, **Scott B**, **Seipke RF**, **Shen B**, **Sherman DH**, **Sivonen K**, **Smanski MJ**, **Sosio M**, **Stegmann E**, **Süssmuth RD**, **Tahlan K**, **Thomas CM**, **Tang Y**, **Truman AW**, **Viaud M**, **Walton JD**, **Walsh CT**, **Weber T**, **van Wezel GP**, **Wilkinson B**, **Willey JM**, **Wohlleben W**, **Wright GD**, **Ziemert N**, **Zhang C**, **Zotchev SB**, **Breitling R**, **Takano E**, **Glöckner FO**. 2015. Minimum Information about a Biosynthetic Gene cluster. Nat Chem Biol **11**:625–631.

2. **Wishart DS**, **Feunang YD**, **Guo AC**, **Lo EJ**, **Marcu A**, **Grant JR**, **Sajed T**, **Johnson D**, **Li C**, **Sayeeda Z**, **Assempour N**, **Iynkkaran I**, **Liu Y**, **Maciejewski A**, **Gale N**, **Wilson A**, **Chin L**, **Cummings R**, **Le D**, **Pon A**, **Knox C**, **Wilson M**. 2018. DrugBank 5.0: a major update to the DrugBank database for 2018. Nucleic Acids Res **46**:D1074–D1082.

3. **Kuhn M**, **Letunic I**, **Jensen LJ**, **Bork P**. 2016. The SIDER database of drugs and side effects. Nucleic Acids Res **44**:D1075–9.

4. **Kidd BA**, **Wroblewska A**, **Boland MR**, **Agudo J**, **Merad M**, **Tatonetti NP**, **Brown**

BD, **Dudley JT**. 2016. Mapping the effects of drugs on the immune system. Nat Biotechnol **34**:47–54.

5. **Boström J**, **Hogner A**, **Schmitt S**. 2006. Do structurally similar ligands bind in a similar fashion? J Med Chem **49**:6716–6725.

6. **Duan Q**, **Reid SP**, **Clark NR**, **Wang Z**, **Fernandez NF**, **Rouillard AD**, **Readhead B**, **Tritsch SR**, **Hodos R**, **Hafner M**, **Niepel M**, **Sorger PK**, **Dudley JT**, **Bavari S**, **Panchal RG**, **Ma'ayan A**. 2016. L1000CDS2: LINCS L1000 characteristic direction signatures search engine. NPJ Syst Biol Appl **2**:257.

7. **Pearson WR**, **Lipman DJ**. 1988. Improved tools for biological sequence comparison. Proc Natl Acad Sci USA **85**:2444–2448.

8. **Pence HE**, **Williams A**. 2010. ChemSpider: An Online Chemical Information Resource. Journal of Chemical Education **87**:1123–1124.

9. **Willett P**, **Barnard JM**, **Downs GM**. 1998. Chemical Similarity Searching. Journal of Chemical Information and Computer Sciences **38**:983–996.

10. **O'Boyle NM**, **Morley C**, **Hutchison GR**. 2008. Pybel: a Python wrapper for the OpenBabel cheminformatics toolkit. Chemistry Central Journal **2**:5.

11. **Li G**, **Ma Q**, **Tang H**, **Paterson AH**, **Xu Y**. 2009. QUBIC: a qualitative biclustering algorithm for analyses of gene expression data. Nucleic Acids Res **37**:e101–e101.

12. **Wang J**, **Jia H**. 2016. Metagenome-wide association studies: fine-mining the microbiome. Nat Rev Microbiol **14**:508–522.

13. **Karlsson FH**, **Tremaroli V**, **Nookaew I**, **Bergström G**, **Behre CJ**, **Fagerberg B**,

Nielsen J, **Bäckhed F**. 2013. Gut metagenome in European women with normal, impaired and diabetic glucose control. Nature **498**:99–103.

14. **Vetizou M**, **Pitt JM**, **Daillere R**, **Lepage P**, **Waldschmitt N**, **Flament C**, **Rusakiewicz S**, **Routy B**, **Roberti MP**, **Duong CPM**, **Poirier-Colame V**, **Roux A**, **Becharef S**, **Formenti S**, **Golden E**, **Cording S**, **Eberl G**, **Schlitzer A**, **Ginhoux F**, **Mani S**, **Yamazaki T**, **Jacquelot N**, **Enot DP**, **Berard M**, **Nigou J**, **Opolon P**, **Eggermont A**, **Woerther PL**, **Chachaty E**, **Chaput N**, **Robert C**, **Mateus C**, **Kroemer G**, **Raoult D**, **Boneca IG**, **Carbonnel F**, **Chamaillard M**, **Zitvogel L**. 2015. Anticancer immunotherapy by CTLA-4 blockade relies on the gut microbiota. Science **350**:1079–1084.

15. **Sivan A**, **Corrales L**, **Hubert N**, **Williams JB**, **Aquino-Michaels K**, **Earley ZM**, **Benyamin FW**, **Lei YM**, **Jabri B**, **Alegre M-L**, **Chang EB**, **Gajewski TF**. 2015. Commensal Bifidobacterium promotes antitumor immunity and facilitates anti-PD-L1 efficacy. Science **350**:1084–1089.

16. **Robinson MD**, **McCarthy DJ**, **Smyth GK**. 2010. edgeR: a Bioconductor package for differential expression analysis of digital gene expression data. Bioinformatics **26**:139–140.

17. **Gu Z**, **Gu L**, **Eils R**, **Schlesner M**, **Brors B**. 2014. circlize implements and enhances circular visualization in R. Bioinformatics **30**:2811–2812.

18. **Shannon P**, **Markiel A**, **Ozier O**, **Baliga NS**, **Wang JT**, **Ramage D**, **Amin N**, **Schwikowski B**, **Ideker T**. 2003. Cytoscape: a software environment for integrated models of biomolecular interaction networks. Genome Res **13**:2498–2504.

19. **Langmead B**, **Salzberg SL**. 2012. Fast gapped-read alignment with Bowtie 2. Nat

Methods **9**:357–359.

20. **Li H**, **Handsaker B**, **Wysoker A**, **Fennell T**, **Ruan J**, **Homer N**, **Marth G**, **Abecasis G**, **Durbin R**, **1000 Genome Project Data Processing Subgroup**. 2009. The Sequence Alignment/Map format and SAMtools. Bioinformatics **25**:2078–2079.

21. **Caporaso JG**, **Kuczynski J**, **Stombaugh J**, **Bittinger K**, **Bushman FD**, **Costello EK**, **Fierer N**, **Peña AG**, **Goodrich JK**, **Gordon JI**, **Huttley GA**, **Kelley ST**, **Knights D**, **Koenig JE**, **Ley RE**, **Lozupone CA**, **McDonald D**, **Muegge BD**, **Pirrung M**, **Reeder J**, **Sevinsky JR**, **Turnbaugh PJ**, **Walters WA**, **Widmann J**, **Yatsunenko T**, **Zaneveld J**, **Knight R**. 2010. QIIME allows analysis of high-throughput community sequencing data. Nat Methods **7**:335–336.
